# Brine available two-dimensional nano-architectonics of fluorescent probe based on phosphate doped ZIF-L for detection of Fe^3+^

**DOI:** 10.1016/j.heliyon.2023.e17884

**Published:** 2023-07-04

**Authors:** Xiaoyun Liu, Xiaoze Wang, Chunyan Sun, Xiaofeng Hu, Weijun Song

**Affiliations:** School of Chemical Engineering, Qinghai University, Xining, 810016, PR China

**Keywords:** Zeolitic imidazolate framework, Iron ion, Fluorescence probe

## Abstract

Herein, we propose a simple and effective strategy for designing a zeolitic imidazolate frameworks (ZIFs) fluorescent probe with a two-dimensional leaf-like structure. By doping ZIF-L with phosphate, we developed a fluorescent probe for iron (Fe^3+^) in systems with high salinity. The fluorescence of P-ZIF-L was quenched effectively with the presence of Fe^3+^. The physicochemical structure, surface morphology, selectivity, stability and composition of the probe were investigated. Under optimized conditions, the fluorescent probe had a detection limit of 0.5 μM. Furthermore, the results that the probe exhibited desirable salt-tolerance and was suitable for determination of Fe^3+^ in brine water samples with satisfactory results.

## 1 Introduction

As one of the most abundant metal elements on the earth, Fe3+ has a special geochemical significance and plays an irreplaceable role in the process of supergene geology and geochemistry due to the higher redox sensitivity compared to that of other metal elements [1]. Fe3+ is now recognized to be important in regulating the magnitude and dynamics of ocean primary productivity, becoming an integral component of the oceans’ biogeochemical cycles. Meanwhile, for the microbes that lived in highly saline environments such as salt lakes, due to the high osmotic pressure, Fe3+ plays a significant key role in biochemical processes at the cellular level of organisms, impacting the structure and activity maintenance of enzymes and proteins, oxygen absorption, transportation and metabolism [2,3]. However, excessive Fe3+ levels in the human body cause arteriosclerosis, and physiological and metabolic disorders [4]. Therefore, an accurate determination of Fe3+ is highly crucial to recording the oxidation state and transformation processes of the atmosphere, oceans and land in the history of the earth as well as to better understand the composition, structure, metabolism and function of uncultured extremophiles in brine. Currently, various technique, mainly including atomic absorption spectrometry (AAS) [5], capillary electrophoresis and inductively coupled plasma emission spectroscopy [6], have been developed for Fe3+ detection. However, due to the complex components of brine and the low concentration of Fe3+ in brine, conventional methods encounter issues, such as expensive large-scale instruments, damage to devices, long experimental cycles, and complicated sample preprocessing. Furthermore, owing to the coexistence of interfering metal ions and low detection specificity and sensitivity of these methods, the conventional methods cannot meet the practical requirements.

Fluorescence detection has been gaining popularity in the way of Fe^3+^ concentration analysis due to its precision, sensitivity, convenience, and real-time feedback [7]. Many materials could be used as Fe^3+^ fluorescent probes, including carbon quantum dots, quantum dots, nanoparticles and metal-organic frameworks [[8], [9], [10], [11], [12], [13], [14], [15], [16], [17], [18], [19], [20], [21]]. Among these, luminescent metal-organic frameworks (LMOFs) have been used as outstanding fluorescent probes due to their adjustable structure, modifiable pores, and easy to bind with specific guest molecules, resulting in luminescence enhancement and quenching [[22], [23], [24]]. Zeolitic imidazolate frameworks (ZIFs) is a kind of LMOFs with simple synthesis, controllable size and excellent biocompatibility [24], imidazole nitrogen sites in porous ZIFs play a key role in molecular recognition to make it could be a candidate as fluorescent probe [25]. As a pseudo-polymorphic of ZIF-8 [26], ZIF-L had same metal ions and organic ligands of ZIF-8 (Zn2+ and 2-methylimidazole), however, it differs in terms of proportion and treatment methods during the preparation process. Most studies on fluorescent probes have focused their attention to the three-dimensional ZIF-8 [[27], [28], [29], [30]]. Because of the weak luminescence and fluorescence properties, ZIF-L always was neglected and hasn't attracted enough attention. Moreover, lower stability also limited the application of ZIFs in aqueous solution as a fluorescent probe [26]. Thus, to improve the stability of ZIFs in aqueous solutions, various methods have been developed. One such method includes using a shell-ligand-exchange reaction to transform the interior surface of layered ZIFs from hydrophilic to hydrophobic [31,32].

**Fig. 1:**
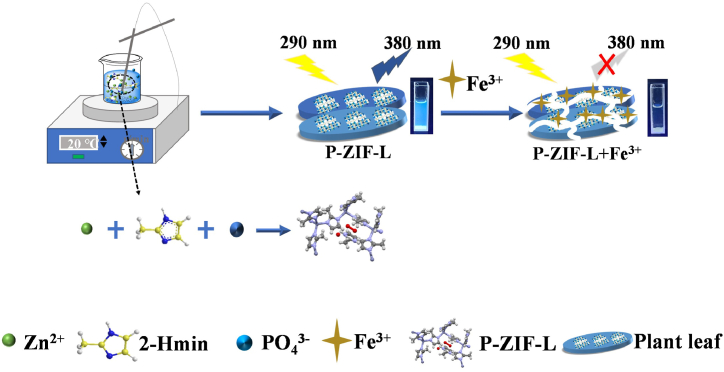
Synthetic procedure of P-ZIF-L and the schematic diagram for Fe^3+^detection.

In this work, a relatively higher fluorescence, hydrophilic and salt-tolerant phosphate doping ZIF-L fluorescent probe (P-ZIF-L) was prepared by adopting a suitable approach at room temperature. The fluorescence at 377 nm (the characteristic emission peaks of P-ZIF-L) was obviously quenched with great selectivity and with a wide linear range in the presence of Fe^3+^, as shown in Fig. 1. The synthesized P-ZIF-L exhibited desirable salt-acid-base resistance, hydrothermal stability and low detection limit. Experimental results showed that its fluorescence (FL) intensity had no obvious changes in a high salt concentration (100 g/L) solution, the leaf-like crystals of P-ZIF-L maintained their structural integrity after hydrothermal treatment for 24 h at 100 °C and remained stable in the solution with pH range from 3 to 11. Furthermore, the phosphate doping made it more hydrophilic and consequently slowed the settlement behavior of P-ZIF-L probe in an aqueous solution. The desirable performance of the P-ZIF-L probe not only decreased the limit of quantitation for Fe^3+^ but also widened its application range, showing great potential for applications in the extreme environmental monitoring.

## 2 Experimental section

### 2.1 Materials and chemicals

Zinc nitrate hexahydrate (Zn(NO_3_)_2·_6H_2_O,>99%) was purchased from Shuangchuan Chemical Reagent Factory (Tianjin, China). Methanol (CH_2_OH, chromatographic grade≥99%）was supplied by Macklin (Shanghai, China). Further, 2-methylimidazole (C_4_H_6_N_2_, >99%), NiCl_2_, KCl, SrCl_2_, CdCl_2_, Pb(NO_3_)_2_, CuCl_2_, CoCl_2_, RbCl, MgCl_2_, CaCl_2_, HgCl_2_, NaCl, CrCl_3_, FeCl_3_, CsCl and Na_3_PO_4_ were purchased from Aladdin Co., Ltd (Shanghai, China). Salt Lake brine was collected from Qarhan Salt Lake. All reagents of analytical grade were used without any additional purification except as noted. The water used in the test was obtained using the AXLK1820-2 ultra-pure water system (Chongqing, China).

### 2.2 Instruments

The stirring process was performed using a magnetic stirrer (DWB Ms7-H550-S, DWB, China). The structure and morphology were characterized by a scanning electron microscope (SEM) (TM4000, HITACHI, Tokyo, Japan). X-ray powder diffraction (XRD) patterns were obtained using an X-ray diffractometer (Rigaku, Kyoto, Japan). The chemical compositions of the materials were measured using an X-ray photoelectron spectrometer (XPS) (TMESCALABTM 250Xi, Termo Scientifific, Waltham, MA, USA). To investigate the functional groups in materials, the infrared spectra in KBr were obtained using a Fourier transform infrared spectroscopy (FTIR, BXII, PerkinElmer, USA). The UV/Vis spectrum was determined by a spectro-photometer (T6, Pu-Analysis General Co., Ltd., China and Cary series UV–vis–NIR, Agilent Technologies, Inc.). Fluorescence spectra were recorded on a fluorescent spectrophotometer (FL-7100, Hitachi, Japan). Photographs were obtained with a dark box type quadruple UV analyser (WFH–203C, Shanghai Jingke Industrial Co., Ltd., China). The contact angle (CA) was measured by an optical contact angle measuring instrument (Zhongchen Instruments Co., Ltd., China). The N_2_ adsorption experiment was performed using by a Specific Surface & Pore Size Analyzer (BEISHIDE Instrument TechnologyCo., Ltd., China).

### 2.3 Synthesis of P-ZIF-L

In this work, P-ZIF-L were synthesized by a simple modified method based on a previously reported ZIF-L synthesis method [33]. Typically, Zn(NO_3_)_2·_6H_2_O (3.96 mM) and 2-methylimidazole (Hmim) (23.38 mM) were dissolved in 80 mL of phosphate solution (1 mg/mL Na_3_PO_3_) respectively. Subsequently, the Zn(NO_3_)_2·_6H_2_O solution was slowly dropped into the Hmim solution, and the mixture was stirred at a low speed at room temperature for 4 h continuously. The obtained white precipitations were separated through centrifugation at 8000 rpm, for 5 min and washed thrice with methanol. Finally, the P-ZIF-L powder was obtained after drying in the oven at 65 °C overnight.

For comparison, the ZIF-L was also prepared according to the previous reported method [33].

### 2.4 Fluorescence detection of Fe^3+^

The fluorescence detection of Fe^3+^ was performed using a fluorescence spectrophotometer at room temperature. The slit width was 5 nm, the scan rate was 2400 nm/min and the photomultiplier tube voltage was 600V. Since fluorescence detection was performed in the aqueous phase, 0.1 g of P-ZIF-L was first fully scattered into 100 mL of deionized water using an ultrasonic mixer, then 1 μL of different Fe^3+^ concentrations were added to 2 mL of P-ZIF-L aqueous dispersion and then transferred to a quartz cell for detection after incubation for 3 min at room temperature. The FL intensity was obtained and recorded at the excitation wavelength of 290 nm.

Probe selectivity studies were conducted under optimal experimental conditions using other metal ions including Ni^2+^, K^+^, Sr^2+^, Cd^2+^, Pb^2+^, Cu^2+^, Co^2+^, Rb^+^, Mg^2+^, Ca^2+^, Hg^2+^, Na^+^, Cr^3+^, Fe^3+^, and Cs^+^. During the experiment, 5 μL of other metal ions (0.1 M) were added to 2 mL of P-ZIF-L (1 g/L) aqueous dispersion successively. As a comparison, the anti-interference ability of P-ZIF-L was further investigated by mixing 10 μL of Fe^3+^ with above various interfering ions.

### 2.5 Evaluation of hydrothermal resistance

To investigate the hydrothermal resistance of samples, 0.2 g of P-ZIF-L power was dispersed into 40 mL of deionized water. Then, the dispersion was transferred into a 100 mL Teflon tank, which was heated in an oven at 100 °C or 150 °C for 24 h. Finally, the sediment obtained after hydrothermally treatment was centrifuged at 10,000 rpm for 15 min and washed thrice by deionized water. Subsequently, the resulting white powder products were dried in the oven for 24 h.

### 2.6 Evaluation of acid and base resistance

To investigate the acid and base resistance of samples, 0.2 g of samples (ZIF-L and P-ZIF-L) was dispersed into 40 mL HCl aqueous solutions with pH values of 3, 5, 9 and 11 respectively. Then, the dispersion was placed in a magnetic stirrer and stirred at room temperature for 24 h. Finally, the sediments after acid or base treatment were centrifuged at 10,000 rpm for 15 min and washed thrice by deionized water. The resultant white powder products were dried in the oven for 24 h.

## 3 Results and discussion

### 3.1 Morphology and microstructure characterizations

**Fig. 2:**
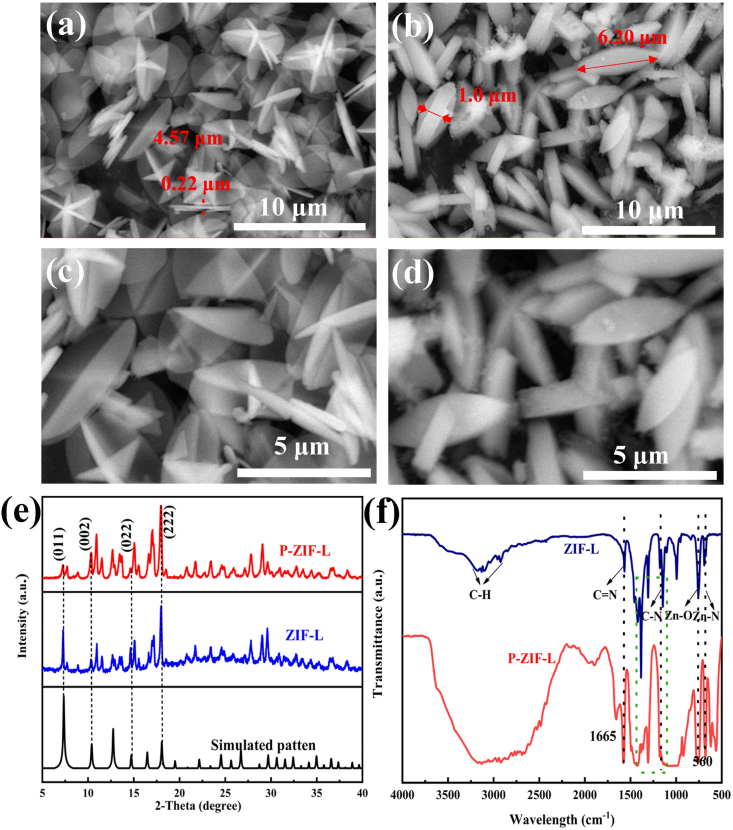
(a, c) SEM images of the ZIF-L and (b, d) P-ZIF-L. (e) XRD of P-ZIF-L, ZIF-L and simulated patterns (f) FT-IR of the P-ZIF-L and ZIF-L.

The as-synthesized P-ZIF-L and ZIF-L powders were investigated by SEM to characterize their microstructure, as shown in Fig. 2. Both powers P-ZIF-L and ZIF-L all showed leaf-like morphology, indicating the efficient synthesis of the two-dimensional ZIF-L. The most obvious difference between P-ZIF-L and ZIF-L was that the former had relatively uncrossed thicker leaf-like crystals in Fig. 2**b** and **d** while the latter had interconnected thinner leaf-like crystals in Fig. 2**a** and **c**. It was speculated that the presence of phosphate restrained priority growth in one direction.

To confirm the crystal structure, P-ZIF-L and ZIF-L were examined using X-ray diffraction (XRD), Fig. 2**e** illustrated the as-synthesized and simulated powder patterns. As shown all peaks of P-ZIF-L and ZIF-L samples in the XRD pattern were sharp and consistent with the patterns of simulated ZIF-8 （CCDS: 823083）, such as 2θ = 7.34° of the (011) plane, 2θ = 10.4° of the (002) plane, 2θ = 12.76° of the (112) plane and 2θ = 18.04° of the (222) plane, confirming that P-ZIF-L was effectively synthesized. The remarkable observation was that the strongest peak in the XRD pattern of P-ZIF-L did not occur at 2θ = 7.34° of the (011) plane but at 2θ = 18.04° of the (222) plane, it might be attributed to the difference in the bridging way of two neighboring sodalite layers, which is the N–H⋯N hydrogen bond from Hmim molecules for ZIF-L and the link of Zn^2+^/mim− (mim− = C_4_H_5_N_2_) ions for ZIF-8 [33]. Different from the patterns of simulated ZIF-8, there were several sub-strong diffraction peaks caused by the crystal structure change, such as 2θ = 15.12°, 17.14°, 29.10°, and 27.86° in P-ZIF-L patterns. Thus, the intricate 2D leaf-like structure of might provide more potential action sites for phosphate doping [33]. No significant changes and typical diffraction peaks of phosphorus species in the XRD pattern of P-ZIF-L were observed due to the low amount of phosphate in the hybrid. Although the location of peaks did not drift, the addition of phosphate affected the height of ZIF-L diffraction peaks, revealing that phosphate was doped into the ZIF-L crystal lattice but not coated on the surface of ZIF-L.

The functional groups in P-ZIF-L were investigated using Fourier transform infrared (FT-IR) spectroscopy, as shown in Fig. 2**f**. since the organic ligand of ZIF-L and P-ZIF-L was Hmim, the absorption peaks of ZIF-L and P-ZIF-L materials in the FT-IR spectra could be attributed primarily to Hmim [34]. Showing characteristic bands of the out-of-plane of the Hmim ring at 600∼800 cm^−1^, the peak at 900∼1350 cm^−1^ was associated with the alkane in-plane bending [35]. The band at 1350∼1500 cm^−1^ was assigned to the entire ring stretching of the imidazole. The aliphatic and aromatic C–H stretching of Hmim was located at 2900∼3200 cm^−1^ [36]. In addition, the absorption peaks at 994 and 1148 cm^−1^ were attributed to the stretching vibration peaks of C–N bonds in imidazole rings, the peaks at 424 cm^−1^ and 690 cm^−1^ were attributed to Zn–N, while the peaks at 1566 cm^−1^, and 2926 cm^−1^ were attributed to C=N, and C–H from ZIF-8, respectively [37]. These proved that the structure of phosphate-doped ZIF-L was the same as that of pure ZIF-L, and that P-ZIF-L was synthesized successfully.

**Fig. 3:**
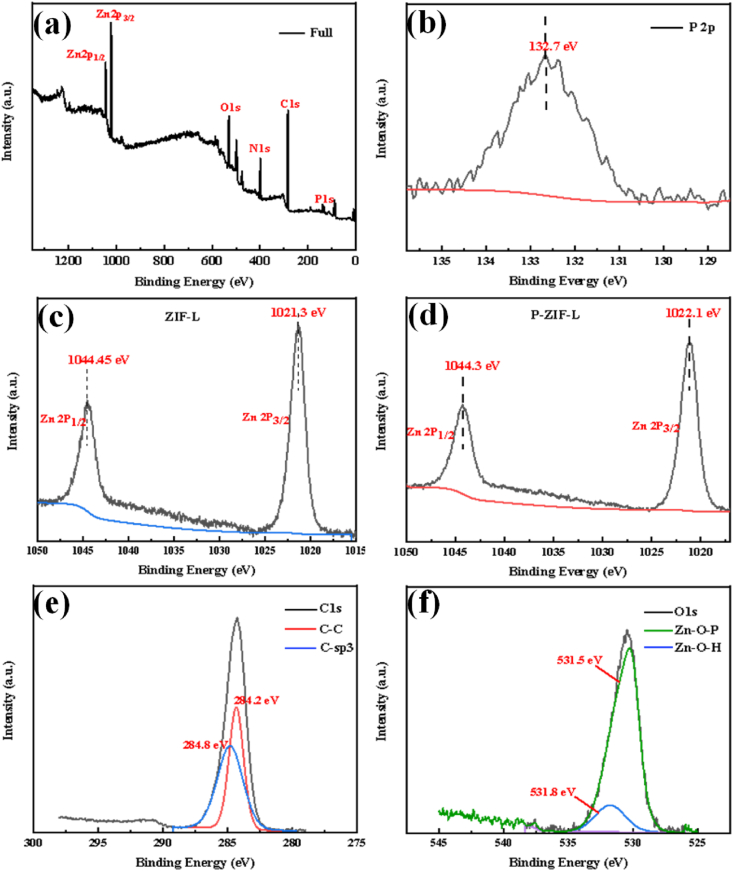
(a) Full XPS spectra of the P-ZIF-L; high-resolution spectra of P-ZIF-L: (b) P2p, (d) Zn2p, (e) C1s and (f) O1s; high-resolution spectra of the ZIF-L: (c) Zn2p.

To further identify the probe composition, X-ray photo electron spectroscopy (XPS) analysis was performed, as shown in Fig. 3**a**, in the range of 0–1300 eV revealed the typical peaks of Zn, O, C, N, and P elements, verified the P elements had been successfully doped into P-ZIF-L. As shown in Fig. 3**b**, the high resolution XPS spectra of P revealed a newly developed P 2p peak at 132.7 eV due to the doped phosphate. However, in comparison with the peak of pure Na_3_PO_4_ (133.1 eV), the P 2p peak exhibited a 0.8 eV shift toward the low-energy side indicating the tight chemical bonding formed between phosphate with ZIF-L [33]. As shown in Fig. 3**c-d**, the Zn 2p spectrum for ZIF-L showed that two peaks were located at 1021.3 eV and 1044.45 eV were attributed to Zn 2p3/2 and 2p 1/2 orbitals, respectively. However, in the Zn 2p spectrum of P-ZIF-L, the locations of two peaks were 1022.1eV (shifted by 0.2 eV) and 1044.3 eV (shifted 0.15 eV), owing to the competition for Zn^2+^ between PO_4_^3−^ and Hmim. Moreover, the C1s spectra presented two peaks at 284.2 eV and 284.6 eV attributed to the presence of C–C and C-sp3 [38], respectively, which were consistent with the C1s peaks of the ZIF-L matrix from the Hmim ligands, as shown in Fig. 3**e**. The O1s spectra of P-ZIF-L that showed an evident decrease in the Zn–OH at the 531.8 eV peak were assigned to the weakened hydroxyl bonding to the Zn metal [39]. Compared with ZIF-L, with Zn–OH groups at the P-ZIF-L surface could dehydrate with phosphate species to generate the Zn–*O*–P/P=O peak at 531.5 eV [40,41]in Fig. 3**f**.

**Fig. 4:**
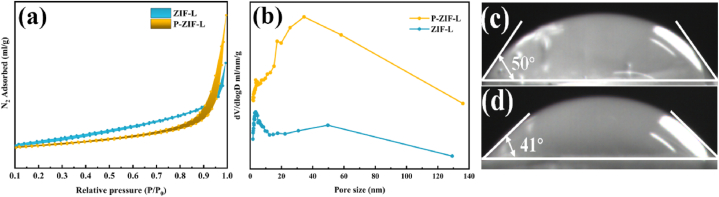
(a) N_2_-adsorption/desorption isotherms ofZIF-L and P-ZIF-L at 77 K. (b) Pore size distribution of ZIF-L and P-ZIF-L. Water contact angle measurements of pellets composed of as synthesized (c) ZIF-Land (d)P-ZIF-L.

To further identify the phosphate that was doped into ZIF-L, N_2_ adsorption for the synthesized ZIF-L and P-ZIF-L were measured at 77.3 K. As shown in Fig. 4**a**, ZIF-L and P-ZIF-L exhibited type-Ⅲ isotherms based on N_2_-adsorption measurement results [42]. It was assumed that ZIF-L was lamellar, stacked structure of leaves. At the beginning the of absorption process, the intermolecular force between adsorbents was strong. During the process of adsorption, the force between the adsorbent and adsorbate became stronger and the adsorption process was accelerated. The isotherm began to increase and N_2_ absorption indicated the presence of micropores [43]. The saturation adsorption volumes with N_2_ were 19.97 mg/L and 12.59 mg/L for ZIF-L and P-ZIF-L, respectively, conforming that phosphate is partially doped into ZIF-L. Fig. 4**b** showed the pore size distribution of P-ZIF-L and ZIF-L. It was evident that most of the pore size of P-ZIF-L was mainly concentrated at 17–80 nm, while most of the pore size of ZIF-L is distributed between 2 and 9 nm. The pore width increased after doping with phosphate. This phenomenon may be due to the combination of unsaturated sites on secondary structural units of phosphate and ZIF-L during encapsulation, making the large crystal slightly [44]. Their corresponding size distribution was presented in Table S1.

To identify the hydrophilicity of the probe, samples of ZIF-L and P-ZIF-L powders were pressed into slices and then the water CA was measured. The results of the measurement are shown in Fig. 4**c** and **d**. The water CA of ZIF-L and P-ZIF-L were 50° and 41° respectively, verifying a mild decline in water CA (about 9°). The hydrophilic behavior of the 2D P-ZIF-L probe was beneficial in improving its dispersion degree and stability in water. It is also consistent with the following experimental results. In addition, the decline of the CA led to a slight increase inits ‘solubility’ the increase in P-ZIF-L FL intensity of P-ZIF-L.

### 3.2 Property characterizations of P-ZIF-L

To evaluate the hydrothermal stability of ZIF-L and P-ZIF-L, the two dispersion samples were treated at 100 °C and 150 °C, respectively, for 24 h. The crystal morphology and structure were characterized using SEM and XRD. As SEM results showed in Fig. S1, the rod-like crystals existed after hydrothermal treatment under 100 °C and 150 °C for ZIF-L, speculating that ZnO was formed [36]. In contrast, P-ZIF-L treated at 150 °C and 100 °C both partly became rod-like as compared to the untreated sample, but leaf-like crystal of P-ZIF-L could still be observed. XRD patterns showed that ZIF-L and P-ZIF-L were treated at 100 °C exhibited characteristic peaks of ZIF-L and the peak intensity was similar to that of P-ZIF-L shown in Fig. S1**e.** This result coincides with the leaf-like shape of SEM. At 150 °C, ZIF-L showed characteristic peaks of ZnO, while the characteristic peaks of ZIF-L decreased observably. Although P-ZIF-L treated at 150 °C also showed characteristic peaks of ZnO, its ZIF-L characteristic peaks did not disappear completely. It could be concluded that P-ZIF-L exhibited better thermal stability than ZIF-L.

Aqueous solutions of different pH values (pH = 3, 5, 9 and 11) were mixed with the ZIF-L and P-ZIF-L powders and stirred for 24 h. The acid-base resistance of the synthesized samples was evaluated by observing the morphology changes and crystal structures of the samples.

The SEM images of the samples after acid-base treatment were given in Fig. S2 The crystal morphology of ZIF-L after pH = 5 treatment was relatively broken, fragmented leaf-like structures could be vaguely observed. The leaf-like shape of P-ZIF-L was still relatively clear, and the upper layer was covered with a part of the salt structure image, which was presumed to be part of phosphate precipitation. When pH = 3, the crystal leaf-like shape of the ZIF-L completely disappeared and was replaced by various of irregularly shaped fragments, while the leaf-like shape of P-ZIF-L was still clear, and the phosphate covered in the upper layer was also reduced, which may be due to the strong acidic condition. Fig. S2**e** shows the XRD pattern of the sample under acid treatment. It could be noted that the crystal morphology after acid treatment did not show the characteristic peak similar to that of ZnO after heat treatment. When pH = 5, the characteristic peaks of pure ZIF-L and P-ZIF-L did not change significantly. Further, the characteristic peaks of ZIF-L and P-ZIF-L did not change significantly after pH = 3 treatments. Thus, P-ZIF-L could maintain its original shape better than ZIF-L under strong acidic conditions. Therefore, P-ZIF-L exhibited excellent acid resistance.

The samples microstructure after treatment in sodium hydroxide solution with pH = 9 and 11 were illustrated in Fig. S3. Similar to the samples after acid treatment, pure ZIF-L was more fragmented under higher alkaline condition of pH = 11, exhibiting fewer leaf-like crystals and more large salt forms. However, under the alkaline condition of pH = 9, the leaf shape of ZIF-L was sufficiently clear, almost similar to that of untreated samples. The P-ZIF-L treated at pH = 11 was slightly blurred, although the leaf shape could be clearly seen. The XRD pattern after alkali treatment is shown in Fig. S3**e**. The intensity of each peak after alkali treatment was enhanced. However, the peak position did not change under pH = 9 or pH = 11. Thus, P-ZIF-L exhibited considerably more effective base resistance than that of ZIF-L.

### 3.3 UV–vis absorption spectra and FL spectra characterizations

To determine the optimal synthesis conditions of P-ZIF-L, a series of single-factor experiments were conducted. Finally, it was determined that the optimal synthesis temperature was 20 °C, the synthesis time was 4 h and the molar ratio was Hmim: Zn (NO3)_2_•6H_2_O = 6:1. More detailed information can be found in the supplementary section, as shown in Fig. S4. Furthermore, to obtain the best analytical performance of the P-ZIF-L, the probe concentration and the dispersion pH were optimized. As shown in Fig. S5**a**, when the probe concentration was 1 g/L, the FL intensity reached a peak value. As depicted in Fig. S5**b**, the FL intensity of P-ZIF-L in alkaline environment was higher than acidic environment. And the intensity reached the maximum value when dispersion pH = 8 [45]. Therefore, the probe concentration of 1 g/L and the dispersion pH of 8 were selected as the optimal testing conditions for the detection of Fe^3+^ in all subsequent experimental processes.

FL intensity increased after phosphate doping as shown in Fig. S6**a**. The image of P-ZIF-L and ZIF-L solid powder under UV light (365 nm) also proved that P-ZIF-L had a higher FL intensity than ZIF-L. Fig. S6**b** showed that FL. Intensity in aqueous P-ZIF-L and ZIF-L shared the same excitation and emission wavelengths（EX = 290 nm，EM = 380 nm）. Fig. S6**c** shows that the UV–vis absorption spectra of P-ZIF-L and ZIF-L exhibited the same region of absorption wavelength from 235 nm to 345 nm; most absorption wavelengths were all at 290 nm, which was in accordance with the optimum excitation wavelength measured by fluorescence photometer. Presumably, doping phosphate to ZIF-L might not affect its absorption of ultraviolet. Furthermore, Fig. S6**d** provided string of emission spectra excited by different wavelengths waves (260–330 nm) of P-ZIF-L, confirmed it had a property of excitation-dependent. FL intensity gradually decreased when the excitation wavelength changed from 290 nm to 320 nm and followed by a gradual redshift in emission wavelength from 375 nm to 405 nm. This phenomenon was caused by two possible reasons. Since the addition of phosphate neither influenced ZIF-L's absorption of ultraviolet, nor changed the optimal emission wavelength, the explanation that the emission wavelength red shift was caused by the interaction between Zn^2+^ and phosphate has been ruled out [46]. Another explanation was that because of some of ZIF-L own wavelength dependence, as previously reported [47], the distinct excitation-dependent shifting tendency occurred in most MOF materials which was really similar to the emitting property of carbon dots and other related semiconductor materials. It might be due to the distinctive structure of these materials with spatial anisotropy, leading to a dimensional confinement effect, forming trapped excitons and excimers with multiple energy levels. Thus, the emission wavelength redshift of P-ZIF-L was primarily attributed to the packed and defective states of the crystal structure.

Fluorescence stability of P-ZIF-L was crucial for its practical function. We prepared experiments on salt tolerance and UV light irradiation time to investigate the fluorescence stability of the P-ZIF-L. The effect of salt (NaCl) concentration on the FL intensity of the P-ZIF-L was investigated in the range of 0–1.8 M (0–100 g/L). As shown in Fig. S7**a**, the FL intensity did not change obviously. This indicated that the high concentration of salt had little effect on the FL intensity of P-ZIF-L, indicating that the probe was highly suitable for the detection of Fe^3+^ in high salt system. In addition, the influence of UV light irradiation time of P-ZIF-L on FL intensity is shown in Fig. S7**b**. The FL intensity remained unchanged over the extension of the UV light irradiation time the FL intensity remain unchanged suggesting that the P-ZIF-L have a good resistance to UV light. All these experimental results indicated that P-ZIF-L exhibited excellent fluorescence stability, could be an effective fluorescent probe for the detection of Fe^3+^ in Salt Lake brine.

### 3.4 Selectivity for detection

**Fig. 5:**
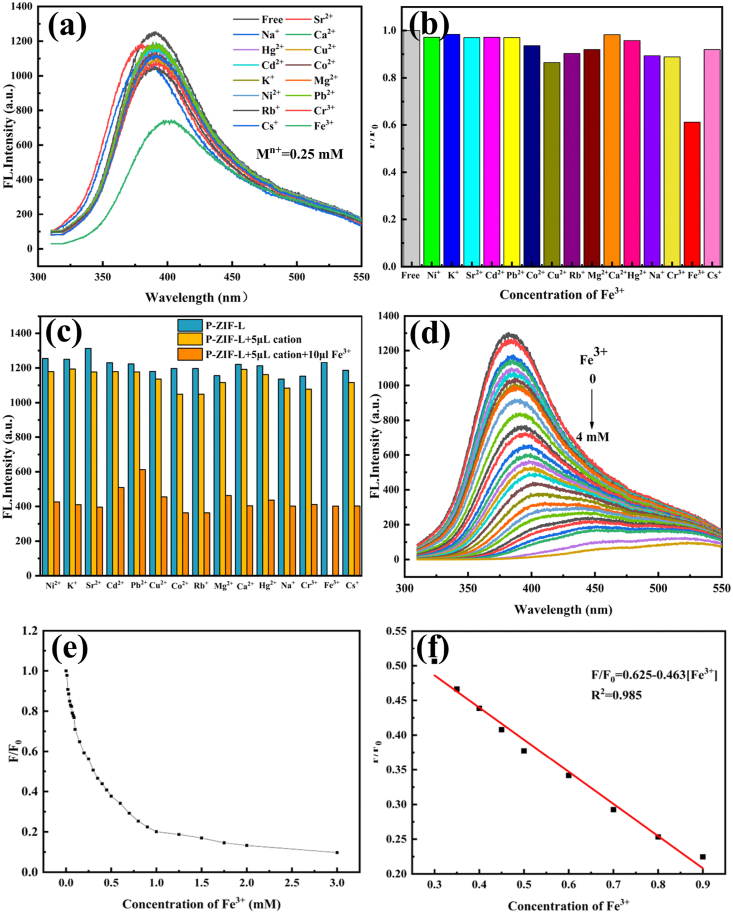
(a) FL spectra of P-ZIF-L (1 g/L) with different metal cations; (b) FL intensity influenced by different metal ions at a concentration of 0.25 mM (F_0_ and F are the FL intensity of the P-ZIF-L without and with metal ions, respectively); (c) the selectivity test of P-ZIF-L (1 g/L) for Fe^3+^ against different metal ions at 290 nm excitation wavelength; (d) FL emission spectra of the P-ZIF-L solutions excited at 290 nm with different concentrations of Fe^3+^ (from up to down: 0–4 mM). The inset displays the corresponding photographs under UV lamp (λ = 300 nm); (e) the relationship between the FL intensity ratio of the P-ZIF-L (F/F_0_) at different concentrations of Fe^3+^ (0–3 mM). (f) The linear equation for the FL quenching ratio at various concentration of Fe^3+^.

It was important for the probe to recognize the target under the interference of other coexisting metal ions. Herein, the probe specificity for recognizing Fe^3+^ was evaluated by interference experiments on P-ZIF-L. Fig. 5**a-b** showed the fluorescence responses of P-ZIF-L in the presence of other metal ions (Ni^2+^, K^+^, Sr^2+^, Cd^2+^, Pb^2+^, Co^2+^, Rb^+^, Cu^2+^, Mg^2+^, Ca^2+^, Hg^2+^, Na^+^, Cr^3+^, Fe^3+^ and Cs^+^; 5 μl of 100 μM metal ions was added into a 2 mL P-ZIF-L dispersion; After these interfering ions were added to the dispersion of P-ZIF-L, no remarkable FL intensity change was observed. However, the addition of Fe^3+^ could considerably decrease the FL intensity of P-ZIF-L, confirming that the P-ZIF-L exhibited excellent recognition ability for Fe^3+^.

In order to further research the anti-interference ability of the P-ZIF-L probe, the anti-interference experiments were conducted, as shown in as Fig. 5**c** when 10 μL of Fe^3+^(100 μM) was added to the dispersion where interfering ions had been added. The FL intensity of the dispersion decrease significantly decreased and the decrease was almost with at the same level. These results indicated that P-ZIF-L could detect Fe^3+^ with high selectivity among the above mentioned 15 cations, especially in the presence of common alkali metal and alkaline earth metal ions in brine systems.

### 3.5 Sensitivity of fluorescence sensing

To further study the sensitivity of the P-ZIF-L for detecting Fe^3+^, the P-ZIF-L samples with different concentrations of Fe^3+^ (0–4 mM) were prepared. The obtained emission spectra for these samples are shown in Fig. 5**d-e**, the FL intensity of P-ZIF-L at 377 nm progressively decreased accompanied by an emission peak shift with increasing Fe^3+^ concentration. Fig. 5**f** gives the linear relationship between the emission intensity ratio (F/F_0_); the results showed that there was a good linear correlation of Fe^3+^ in the range of 300–900 μM （I/I_0_ = 0.625–0.463 [Fe^3+^], R^2^ = 0.985). The -Volmer equation (1) is expressed as follow:

(1) $$I_{0}/I=1+K_{SV}\left[{Fe}^{3+}\right]$$

where K_SV_ was the Stern-Volmer constant. I_0_ and I are the FL intensity in the absence and presence of Fe^3+^, respectively. The detection limit [48] of 0.5 μM was obtained based on a 3δ/b (δ is the standard deviation of the blank sample signal (n = 10) and b is the slope of the linear calibration plot) which was lower than most of the previous reported MOF assays for Fe^3+^ detection. As summarized in Table 1, comparing P-ZIF-L with previous literature that reported MOF assays for the detection of Fe^3+^, the P-ZIF-L showed a wide detection range and low detection limits. The fluorescent technique depends on accurate measurements of signal intensities, which susceptible to interference because of the poor dispersibility of most MOF. To conquer the limitation, we explored a more hydrophilic and more dispersed P-ZIF-L to detect Fe^3+^. A more stable fluorescent signal was output and shows high detection accuracy.

**Table 1:** Comparison of MOF fluorescent probes for the detection of Fe^3+^.

No.	Probe	Linear Range	Detection Limit	Ref.
1	AL-MOF	10–70 μM	6.62 μM	[49]
2	R6G@ZIF-8	0–5 mM	5 μM	[42]
3	HCAA@Uio-66	0–400 mM	4.87 μM	[50]
4	Tb-MOF	0–2 mM	4.84 μM	[51]
5	AL-Zn-ZIF-8	1–10 μM	3.9 μM	[43]
6	Zn(Ⅱ)-MOF	44–64 μM	3.4 μM	[52]
**7**	**P-ZIF-L**	**300**–**900 μM**	**0.5 μM**	**This work**

### 3.6 Fe^3+^ detection in real samples

To evaluate the accuracy and applicability of the P-ZIF-L fluorescent probe in practical applications, spiking and recovery experiments were conducted in tap water and Salt Lake brine respectively. The P-ZIF-L aqueous dispersion without and with the addition of Fe^3+^ was detected by measuring FL spectra. As showed in Table 2, after the addition of Fe^3+^ in the aqueous dispersion of P-ZIF-L, the detected Fe^3+^ was in good agreement with the spiked one, demonstrating high detection recoveries of 93.33%–106.67%. The above results suggested that the P-ZIF-L fluorescence exhibited high accuracy and reliability for the determination of Fe^3+^ in a real sample, particularly in complex brine systems.

**Table 2:** Detection of Fe^3+^ in real samples.

	Spiked amounts (mM)	P-ZIF-L found (mM)	Recovery (%)
Tap water	0	0.5	
	0.3	0.81	103.33
	0.45	0.96	102.22
	0.6	1.14	106.67
Qarhan Salt Lake brine	0	0.03	
	0.3	0.32	96.67
	0.45	0.47	97.78
	0.6	0.59	93.33

### 3.7 Fluorescence detection mechanisms

ZIFs are kinds of MOFs material, which is a coordination polymer with certain size obtained by the molecular assembly method of metal ions and organic ligands. As literature [53] reported, the luminescence mechanism of MOFs could be explained as direct organic ligands excitation (particularly from the highly conjugated ligands), metal-centered emission (widely observed in lanthanide MOFs through the so-called antenna effect), and charge-transfer such as ligand-to-metal charge transfer (LMCT) or metal-to-ligand charge transfer (MLCT). Furthermore, the guest molecules can also result in luminescence onto MOFs. We studied the fluorescence properties both of the dimethylimidazole ligand and the ZIF-L, the strong luminescence of ZIF-L was observed at 370 nm, while the free dimethylimidazole ligand also displays a weak luminescence at 382 nm when excited at 290 nm at room temperature. The fluorescence enhancement and blue shift of ZIF-L were attributed to the formation of the framework structure, which made the two-dimensional skeleton rigid. The intramolecular/intermolecular interaction between the organic linkers was maximized for energy transfer and the energy gap in the ligands is reduced, the emission of Hmim is probably attributed to the π*-π transition of H-mim [54]. Therefore, it is speculated that the mechanism of ZIF-L luminescence is excitation of organic ligands.

According to the previous literature reported [55,56], the fluorescence quenching mechanism by Fe^3+^ usually includes the following possible situation: collapse of the framework, ion exchange, inner filter effect (IFE) phenomenon and some specific interactions between Fe^3+^ and MOF. These possible mechanisms were considered respectively. In this study, when 5 μL of Fe^3+^ solutions were added into the probe dispersion, its fluorescence was immediately decreased, while the probe was stained and did not fade after washing with deionized water. Furthermore, the fluorescence of the used probe could not recover when it was dispersed again. Thus, it could be deduced that fluorescence quenching was possibly caused by framework collapse. To further demonstrate the fluorescence quenching mechanism, another experiment was conducted; in this experiment, the P-ZIF-L probe into 1 mM Fe^3+^ solution for 24 h. Subsequently, the FT-IR spectrum of the probe was tested. The comparison of FT-IR spectra before and after revealed that the characteristic absorption at 600-800 cm^−1^ and 1350-1500 cm^−1^, corresponding to the outer plane bending and inner plane bending in the imidazole ring respectively, showed that the spectra were considerably weakened, and the characteristic absorption peaks at 2900-3200 cm^−1^ almost disappeared in the spectrum of soaked P-ZIF-L. Therefore, the relation of the quenching mechanism to framework collapse had been further verified. Notably, a small peak at 520 cm^−1^, which was attributed to the Fe–O vibration absorption, appeared in the spectrum of soaked P-ZIF-L; this demonstrated that there was some ion exchange between Fe^3+^ and imidazole ring shown in Fig. S8**a**. We also conducted resonance energy transfer test experiments, that is, we obtained the UV absorption spectra of Fe^3+^ and the excitation and emission peaks of P-ZIF-L. As exhibited in Fig. S8**b**, the UV absorption wavelength range of the Fe^3+^ overlapped with the excitation wavelength range of P-ZIF-L indicating that Fe^3+^ could absorb most of the excitation energies effectively and blocked energy transfer path of the ligand thus leading to the fluorescence quenching of P-ZIF-L. Thus, the mechanism of P-ZIF-L's fluorescence quenched by Fe^3+^ might be a result arising out of the combination of three factors including framework collapse, ion exchange and inner filter effect (IFE) phenomenon.

## 4 Conclusions

In summary, P-ZIF-L was synthesized using a simple way of changing solvent water by a sodium phosphate solution during ZIF-L synthesis process. The prepared P-ZIF-L exhibited uncrossed thicker leaf-like crystals with higher FL intensity as compared with that of ZIF-L. The prepared P-ZIF-L could quench Fe^3+^ might be a combining result of three mechanisms. Meanwhile, P-ZIF-L exhibited excellent selectivity, wide detection range, especially the lower detection limit than the previous literature and the experimental data [[49], [50], [51], [52]]. The good salt resistance as well as high hydrothermal stability, acid and base resistance not only widen detection range and fields but also provided a new approach to solve the problem of seriously interfere in Salt Lake brine system. Additionally, since P-ZIF-L has a larger pore size and pore volum, P-ZIF-L could be a great potential candidate mesoporous material with faster mass transfer speed, high activity and stability to be applied to various fields. However, though P-ZIF-L had a smaller contact angle, as well as better dispersion and stability in aqueous solution compared to ZIF-L, the dispersibility and stability of this assay failed to meet expectations, as subsidence may occur later; this might be disadvantageous influence to the detection. Therefore, further studies would be needed to enhance particle-solvent interactions to avoiding agglomeration and sedimentation.

## Author contribution statement

Xiaoyun Liu: Performed the experiments; Analyzed and interpreted the data; Wrote the paper.

Xiaoze Wang, Xiaofeng Hu, Chunyan Sun: Analyzed and interpreted the data.

Weijun Song: Conceived and designed the experiments; Contributed reagents, materials, analysis tools or data.

## Data availability statement

No data was used for the research described in the article.

## Declaration of competing interest

The authors declare the following financial interests/personal relationships which may be considered as potential competing interests: Weijun Song reports financial support was provided by Qinghai Science and Technology Department.
