# Supplementary material for: Brine available two-dimensional nano-architectonics of fluorescent probe based on phosphate doped ZIF-L for detection of Fe3+
Source: Heliyon. 2023 Jul 4;9(7):e17884. doi: 10.1016/j.heliyon.2023.e17884 (PMC10393607; doi:10.1016/j.heliyon.2023.e17884)
Supplement: Multimedia component 1 [file mmc1.docx]

**Supplementary Materials**

**A two-dimensional brine available fluorescent probe based on phosphate doped ZIF-L for detection of Fe^3+^**

1. **property characterizations of P-ZIF-L**

**Table S1**

Pore structure parameters of ZIF-L and P-ZIF-L materials.

| **materials** | **BET(m^2^/g)** | **Langmuir(m^2^/g)** | **Pore volume (cm^3^/g)** | **Pore size (nm)** |
| --- | --- | --- | --- | --- |
| ZIF-L | 17.302 | 32.798 | 0.047 | 10.958 |
| P-ZIF-L | 11.378 | 18.650 | 0.070 | 24.434 |


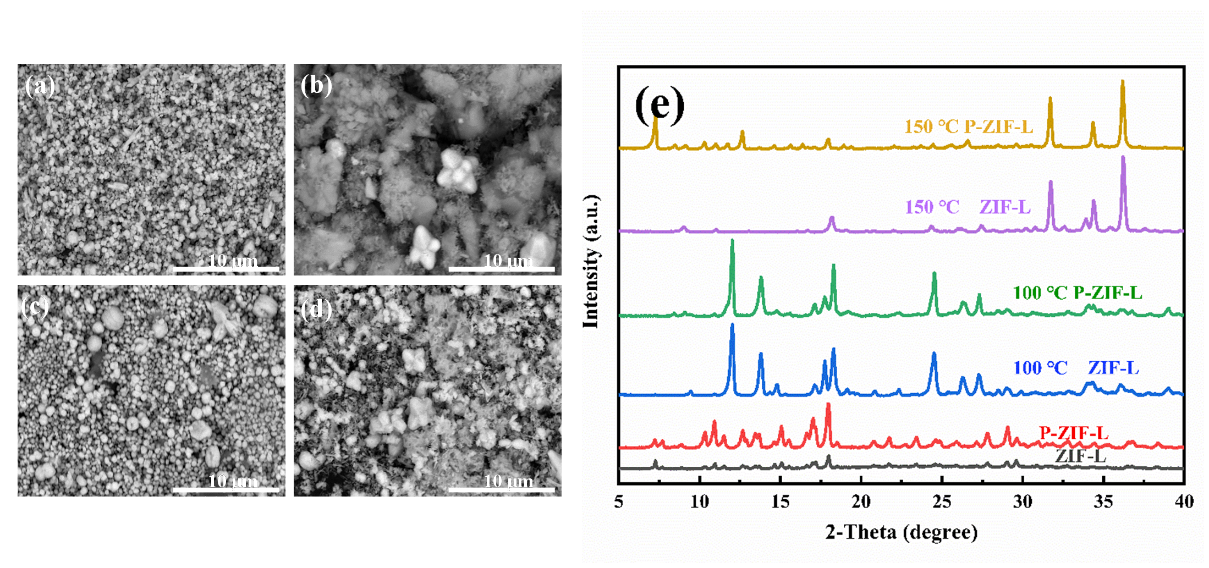


**Fig. S1.** SEM images of ZIF-L following hydrothermal treatment at (a)100°C and (c)150°C; SEM images of P- ZIF-L following a hydrothermal treatment at (b)100°C and (d)150°C. (e)XRD patterns of ZIF-L and P-ZIF-L following hydrothermal treatment at 100℃ and150℃.


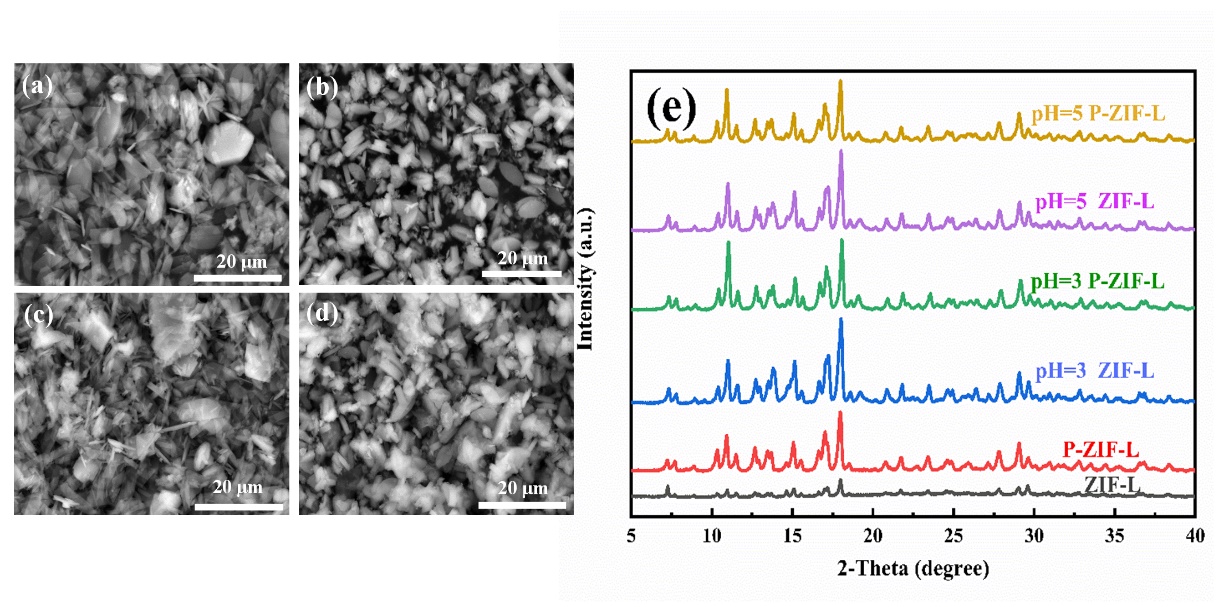


**Fig. S2.** SEM images of ZIF-L following acidic treatment at (a) pH=5 and (c) pH=3; SEM images of P- ZIF-L following acidic treatment at (b) pH=5 and (d) pH=3; (e)XRD patterns of ZIF-L and P-ZIF-L following acidic treatment at pH=5 and pH=3.


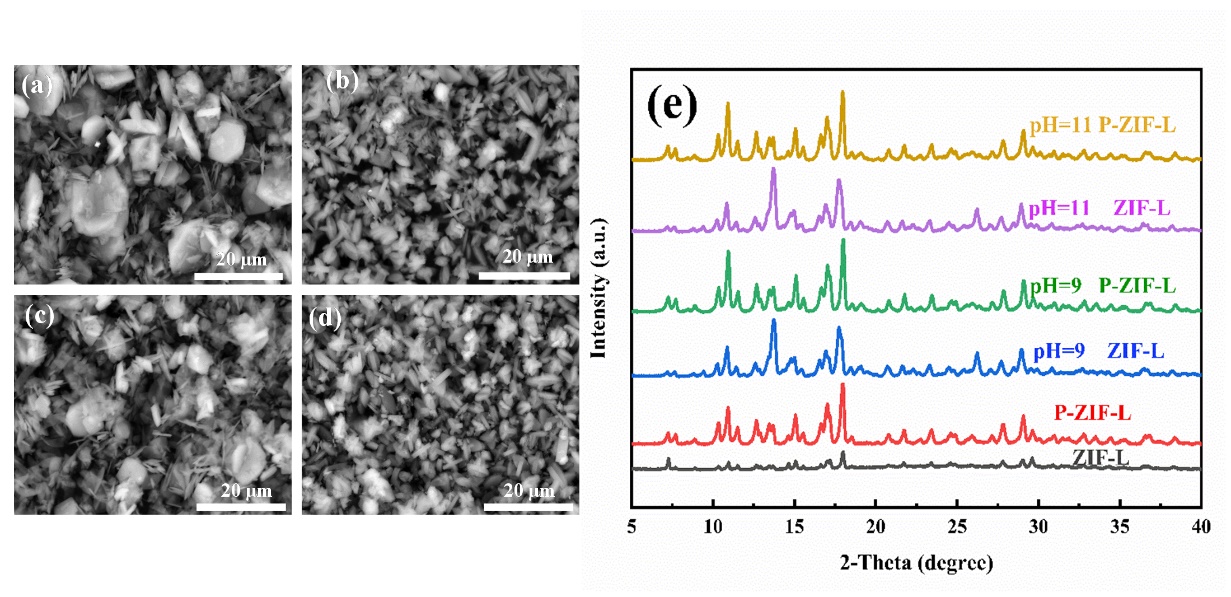


**Fig. S3.** SEM images of ZIF-L following basic treatment at (a) pH=9 and (c) pH=11; SEM images of P- ZIF-L following basic treatment at (b) pH=9 and (d) pH=11; (e)XRD patterns of ZIF-L and P-ZIF-L following basic treatment at pH=9 and pH=11.

1. **Fluorescence condition**

As the standard of the fluorescence intensity of P-ZIF-L (and ZIF-L) under the same test conditions, a single factor variable experiment was designed to explore the synthesis conditions under which the fluorescence intensity of P-ZIF-L (and ZIF-L) was the highest. According to the methods in the previous literature^38^, the raw material ratios of Hmim and Zn(NO_3_)_2_·6H_2_O (1:2, 1:4, 1:6, 1:8, 4:1, 6:1, 8:1) of synthesized ZIF-L were explored, as shown in (Fig. S4**a**). The raw material ratio of Hmim and Zn(NO_3_)_2_·6H_2_O (1:2, 1:4, 1:6, 1:8, 4:1, 6:1, 8:1), reaction temperature (20℃, 30℃, 40℃, 50℃, 60℃), synthesis time (4 h, 5 h, 6 h, 7 h, 8 h ) were considered on the fluorescence intensity of P-ZIF-L respectively, the results are shown in (Fig. S4**b-d)**. As a result, the ratio of raw materials required for the synthesis of P-ZIF-L with the highest fluorescence intensity is still Hmim: Zn(NO_3_)_2_·6H_2_O=6:1. The conditions for the highest fluorescence intensity of synthesized ZIF-L are as follows: Hmim : Zn(NO_3_)_2_·6H_2_O=6:1, synthesis temperature 20℃ (room temperature), synthesis time 4 hours.


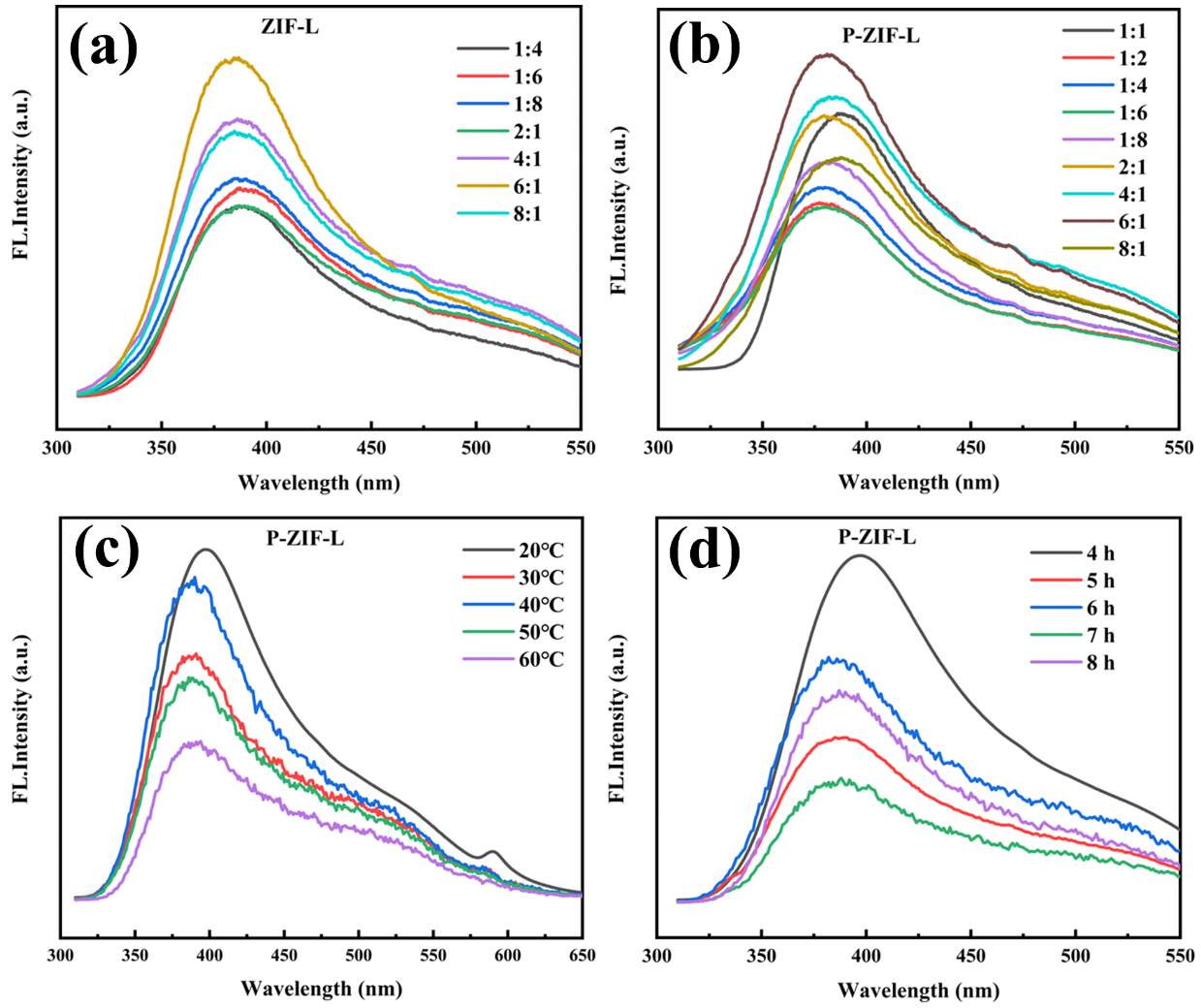


**Fig. S4.** FL spectra of ZIF-L synthesized under different H-mim : Zn(NO_3_)_2_·6H_2_O proportion conditions (a).

FL spectra of P-ZIF-L synthesized under different conditions:

Hmim : Zn(NO_3_)_2_·6H_2_O proportion (b), temperature (c) and time (d);


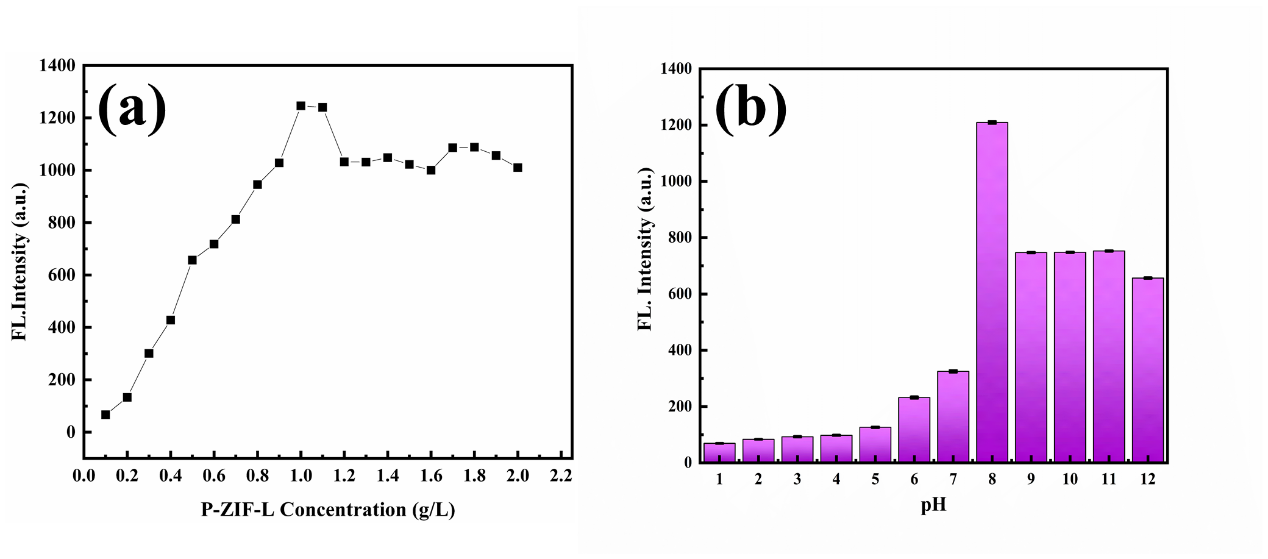


**Fig. S5.** (a) FL intensity of P-ZIF-L at different concentrations; (b) FL intensity of P-ZIF-L at differ


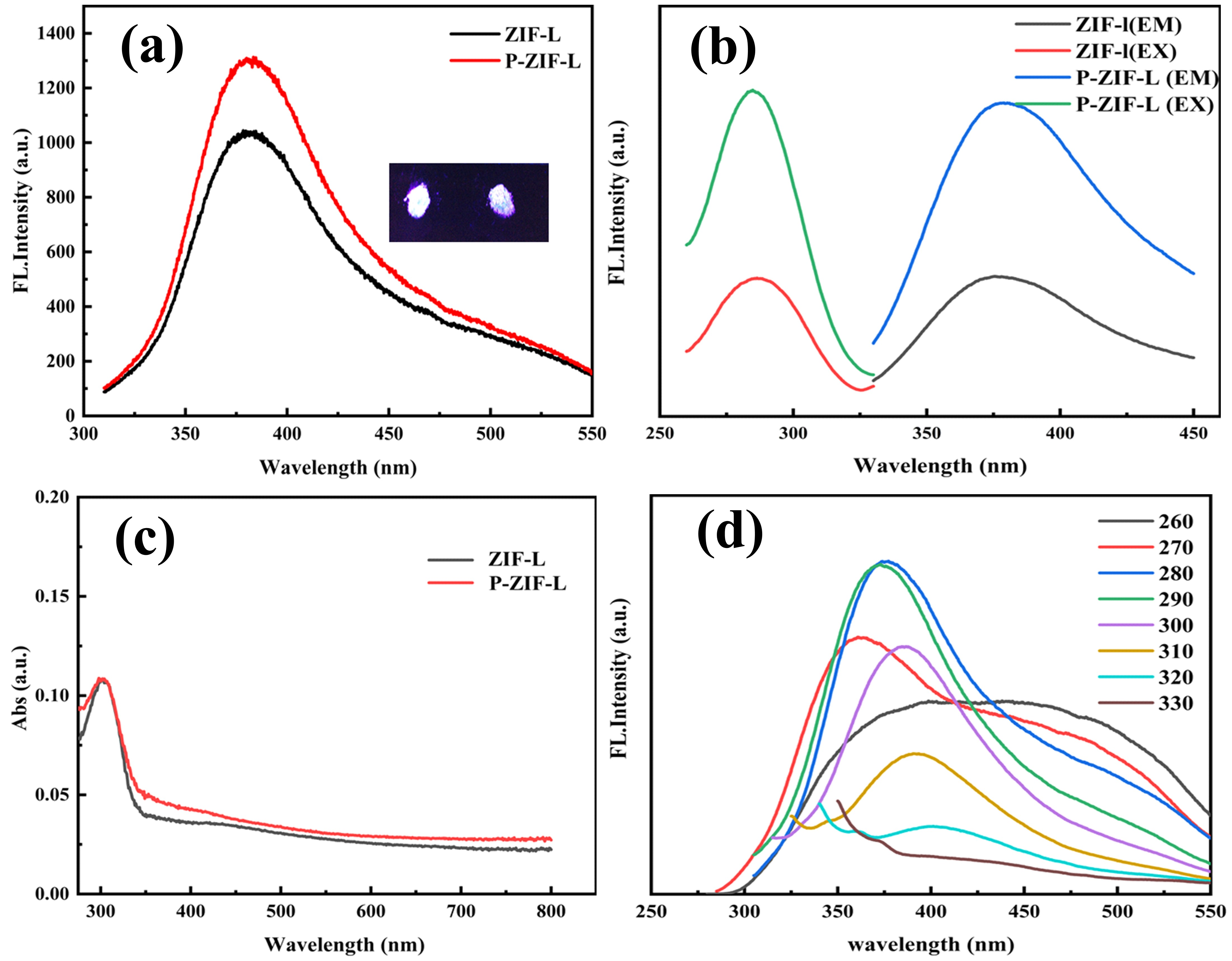


**Fig. S6.** (a) FL spectra intensity of ZIF-L and P-ZIF-L at same conditions, the photographic image of P-ZIF-L(left) and ZIF-L(right) solid powder under visible light and UV light (365 nm). (b) FL spectra of ZIF-L and P-ZIF-L; (c)UV-vis absorption spectra of

ZIF-L and P-ZIF-L; (d) FL spectra of P-ZIF-L at different excitation wavelengths.


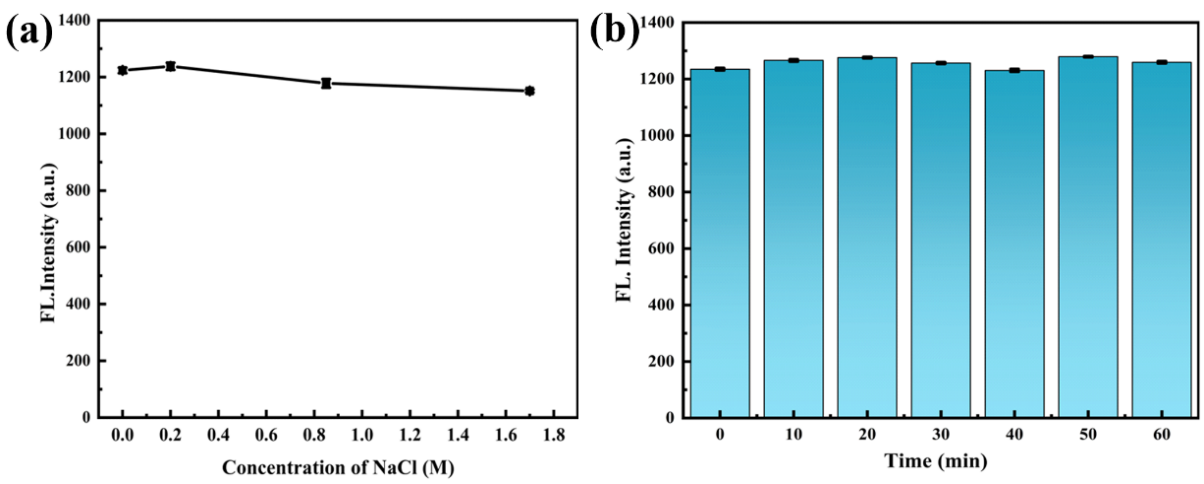


**Fig. S7.** Salt resistance (a) and UV lamp resistance (b) of P-ZIF-L

1. **Fluorescence detection mechanisms**


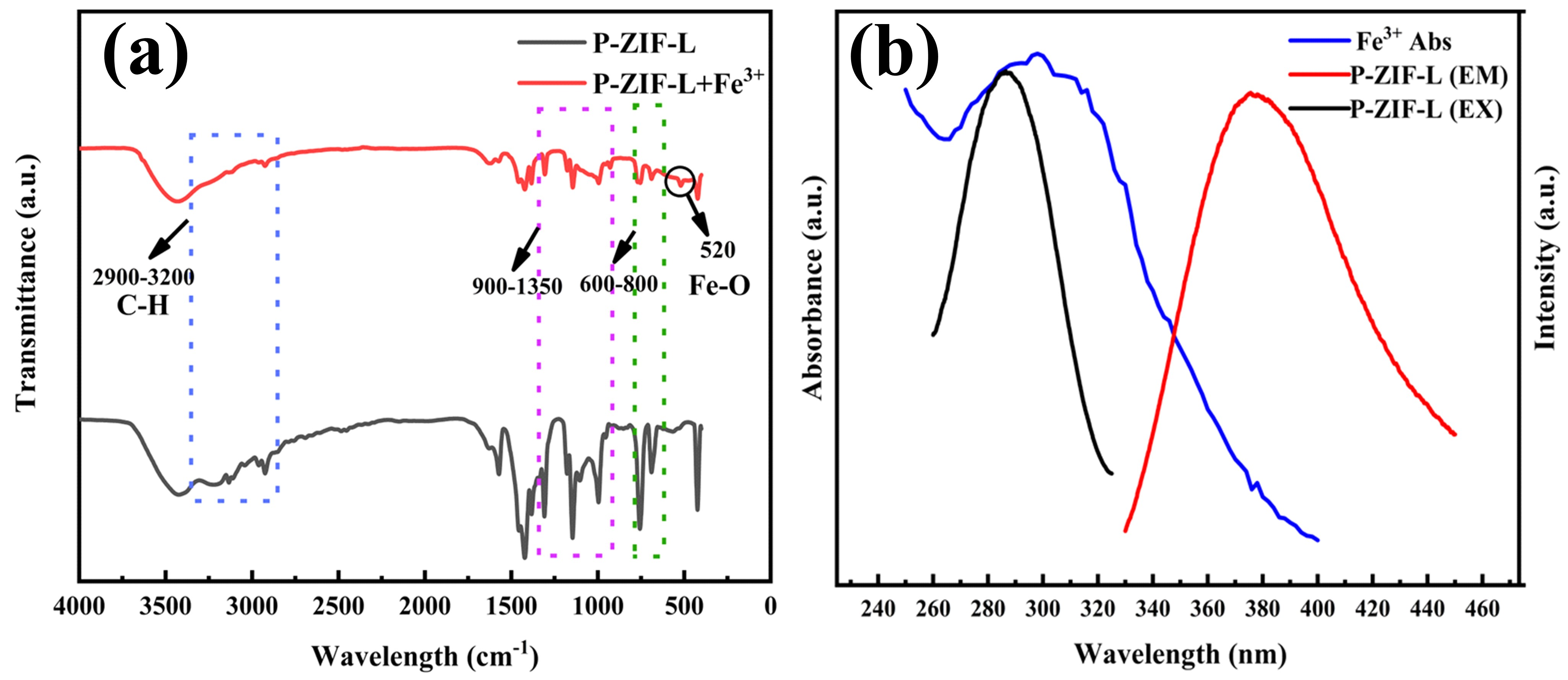


**Fig. S8.** (a) FT-IR spectra of P-ZIF-L and P-ZIF-L+Fe^3+^. (b) UV-Vis absorbance spectra of the Fe^3+^ (blue line) and the excitation/emission spectra of P-ZIF-L. The photos under the Fluorescent.
